# THBS1 and THBS2 Enhance the In Vitro Proliferation, Adhesion, Migration and Invasion of Intrahepatic Cholangiocarcinoma Cells

**DOI:** 10.3390/ijms25031782

**Published:** 2024-02-01

**Authors:** Eleonora Corbella, Claudia Fara, Francesca Covarelli, Veronica Porreca, Biagio Palmisano, Giuseppina Mignogna, Alessandro Corsi, Mara Riminucci, Bruno Maras, Carmine Mancone

**Affiliations:** 1 Department of Molecular Medicine, Sapienza University of Rome, Viale del Policlinico 155, 00161 Rome, Italy; corbella.2016275@studenti.uniroma1.it (E.C.); fara.1915355@studenti.uniroma1.it (C.F.); covarelli.1735136@studenti.uniroma1.it (F.C.); veronicaporreca7@gmail.com (V.P.); alessandro.corsi@uniroma1.it (A.C.); mara.riminucci@uniroma1.it (M.R.); 2 Department of Radiology, Oncology and Pathology, Sapienza University of Rome, Viale del Policlinico 155, 00161 Rome, Italy; biagio.palmisano@uniroma1.it; 3 Department of Biochemistry Science, Sapienza University of Rome, Viale Regina Elena 332, 00185 Rome, Italy; pina.mignogna@uniroma1.it (G.M.); bruno.maras@uniroma1.it (B.M.)

**Keywords:** intrahepatic cholangiocarcinoma, tumor microenvironment, THBS1, THBS2, angiogenesis, lymphangiogenesis

## Abstract

In intrahepatic cholangiocarcinoma (iCCA), thrombospondin 1 (THBS1) and 2 (THBS2) are soluble mediators released in the tumor microenvironment (TME) that contribute to the metastatic spreading of iCCA cells via a lymphatic network by the trans-differentiation of vascular endothelial cells to a lymphatic-like phenotype. To study the direct role of THBS1 and THBS2 on the iCCA cells, well-established epithelial (HuCCT-1) and mesenchymal (CCLP1) iCCA cell lines were subjected to recombinant human THBS1 and THBS2 (rhTHBS1, rhTHBS2) for cellular function assays. Cell growth, cell adhesion, migration, and invasion were all enhanced in both CCLP1 and HuCCT-1 cells by the treatment with either rhTHBS1 or rhTHBS2, although they showed some variability in their intensity of speeding up cellular processes. rhTHBS2 was more intense in inducing invasiveness and in committing the HuCCT-1 cells to a mesenchymal-like phenotype and was therefore a stronger enhancer of the malignant behavior of iCCA cells compared to rhTHBS1. Our data extend the role of THBS1 and THBS2, which are not only able to hinder the vascular network and promote tumor-associated lymphangiogenesis but also exacerbate the malignant behavior of the iCCA cells.

## 1. Introduction

Intrahepatic cholangiocarcinoma (iCCA) is a deadly tumor that exerts its aggressiveness through the high proliferation and invasive abilities of malignant cholangiocytes [1,2]. These abilities lead to a silent and rapid intrahepatic expansion and metastasis in lymph nodes, making it difficult to intervene with curative treatments [3]. The development of a reactive tumor microenvironment (TME) is pivotal in exacerbating the malignant phenotype of iCCA cells [4,5]. Increased attention has been focused on soluble mediators (growth factors, matricellular proteins, cytokines, and morphogens) released in the iCCA TME by non-cancerous cells able to enhance the malignant phenotype of the iCCA cells [1,6,7,8,9].

In this research field, we recently found that the matricellular proteins thrombospondin 1 (THBS1) and 2 (THBS2) are highly expressed and released in the extracellular fluid (ECF) of the iCCA TME from cancer-associated fibroblasts (CAFs) [10]. The thrombospondins are a family of matricellular glycoproteins made up of five members further divided into two subgroups taking into account their structural differences [11]. THBS1 and THBS2 belong to the first subgroup, both having a trimeric structure with three identical subunits of about 180 kDa. Each subunit is made up of N- and C-terminal globular domains connected by a central stalk composed of the procollagen domain, and a series of repeated domains: three type 1 repeats (TSR1s), three type 2 repeats (TSR2s), and seven type 3 repeats (TSR3s) [11]. These multifunctional proteins are known to inhibit angiogenesis by affecting cell motility, apoptosis, and proliferation of vascular endothelial cells [12]. We demonstrated that, in the frame of iCCA, THBS1 and THBS2, along with pigment epithelium-derived factor (PEDF), another potent inhibitor of angiogenesis, indeed inhibited the blood vessel formation, but at the same time, they promoted the trans-differentiation of vascular endothelial cells to a lymphatic-like phenotype [10]. Most importantly, in mouse xenograft experiments with CCLP1, an established human cell line of iCCA, the simultaneous presence of blocking antibodies for THBS1, THBS2, and PEDF in the iCCA ECF was able to drastically reduce the tumor growth and the extent of tumor infiltration in local lymph nodes. PEDF is generally considered a tumor suppressor, while for THBS1 and THBS2, controversial roles in cancer have been reported [13,14]. In some kinds of tumors, both THBS1 and THBS2 showed oncogenic properties by promoting cell migration and invasion [15,16,17,18], while in others, the two isoforms showed conflicting roles [19,20,21]. It is conceivable that the anti- or pro-tumorigenic roles of THBS1 and THBS2 may depend on the molecular and cellular composition of the TME but also that each of the thrombospondins may activate different signaling pathways. In this paper, we addressed the specific roles of each thrombospondin in priming cellular processes involved in the malignancy of iCCA cells. We found that although both the THBS1 and THBS2 exacerbate the malignant behavior of iCCA cells, they showed some variability in their intensity of speeding up cellular processes involved in the pathobiology of iCCA.

## 2. Results

### 2.1. Recombinant THBS1 and THBS2 Enhance Colony Formation in CCLP1 Cells

**Figure 1:**
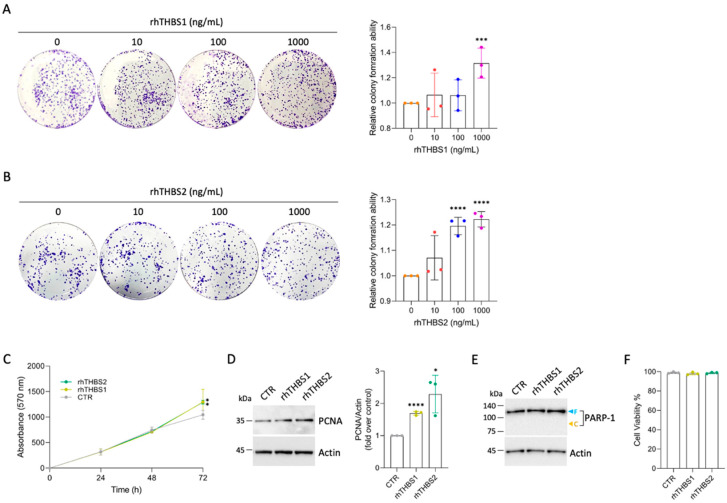
rhTHBS1 and rhTHBS2 increase colony formation in CCLP1 cell line. Clonogenic assays performed with CCLP1 cells in the presence of rhTHBS1 (**A**) and rhTHBS2 (**B**) at the indicated concentrations. (**C**) Time course of CCLP1 cell growth assessed using the MTT cell viability assay at various time points in the presence of 1000 ng/mL of rhTHBS1 and rhTHBS2. (**D**) Western blot analysis for PCNA on whole-cell extracts of CCLP1 cells cultured in the presence of 1000 ng/mL of rhTHBS1 and rhTHBS2 for 48 h; actin was used as a loading control. Quantification from three independent immunoblots by densitometry analysis is shown. (**E**) Western blot analysis for PARP-1 on whole-cell extracts of CCLP1 cells cultured in the presence of 1000 ng/mL of rhTHBS1 and rhTHBS2 for 48 h. Arrows indicate the expected molecular weights for the full-length (blue) and cleaved (yellow) forms; actin was used as a loading control. (**F**) Determination of cell viability after treating CCLP1 cells with 1000 ng/mL of rhTHBS1 and rhTHBS2. The cell viability was measured after 8 h by the intake of trypan blue by dead cells; * *p* < 0.05; *** *p* < 0.001, **** *p* < 0.0001 (*t*-test).

To assess the role of each of the two thrombospondins in the cellular processes that marked the progression of iCCA, we treated the CCLP1, an established human cell line of iCCA, with either human THBS1 or THBS2 in their recombinant form (rhTHBS1 and rhTHBS2). We first studied the role of rhTHBS1 and rhTHBS2 on CCLP1 cell growth. To this aim, clonogenic assays were performed subjecting CCLP1 cells to increasing concentrations of rhTHBS1 or rhTHBS2. A statistically significant increase in the number of colonies was observed 7 days after the treatment with either rhTHBS1 or rhTHBS2 up to 1000 ng/mL dose (Figure 1A,B).

Treatment with rhTHBS2 at 100 ng/mL promoted colony formation just below the number observed in the rhTHBS1 experiment only at 1000 ng/mL (Figure 1B). It should be noted that the concentration of 100 ng/mL of THBS2 is found in human serum, while the concentration of THBS1 is higher by an order of magnitude [10]. The time course of CCLP1 growth, assessed by the MTT cell viability assay, showed increased cell growth in the presence of 1000 ng/mL of rhTHBS1 and rhTHBS2 (Figure 1C). Moreover, the proliferating cell nuclear antigen (PCNA) expression quantified by Western blot analysis was found to be increased (Figure 1D). Neither of the two thrombospondins at the concentrations utilized in the experiments induced the formation of the apoptotic marker Poly(ADP-ribose) polymerase 1 (PARP-1) cleavage fragment (Figure 1E) nor changes in cell viability (Figure 1F).

### 2.2. rhTHBS1 and rhTHBS2 Promote CCLP1 Cell Adhesion, Migration, and Invasion

**Figure 2:**
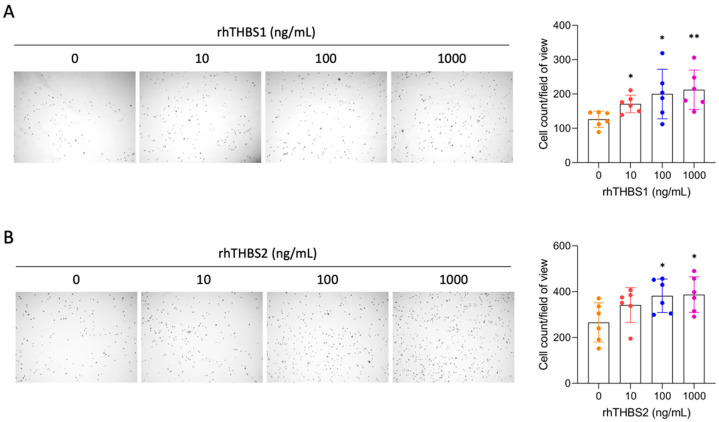
rhTHBS1 and rhTHBS2 enhance CCLP1 cell adhesion. Impact on cellular attachment in CCLP1 cells in response to varying concentrations of rhTHBS1 (**A**) and rhTHBS2 (**B**); magnification 4×. Mean values (±SD) of 6 independent repeats are shown; * *p* < 0.05; ** *p* < 0.01 (*t*-test).

Since many studies demonstrated that THBS1 and THBS2 mediate cellular adhesion of numerous cell types [14,15,16,17,18], we explored changes in cellular adhesive properties induced by rhTHBS1 and rhTHBS2 in iCCA cells. We performed a cell adhesion assay subjecting CCLP1 cells to increasing concentrations of either rhTHBS1 or rhTHBS2. We observed a statistically significant increase in the attachment of CCLP1 cells upon either rhTHBS1 or rhTHBS2 treatment (Figure 2A,B).

**Figure 3:**
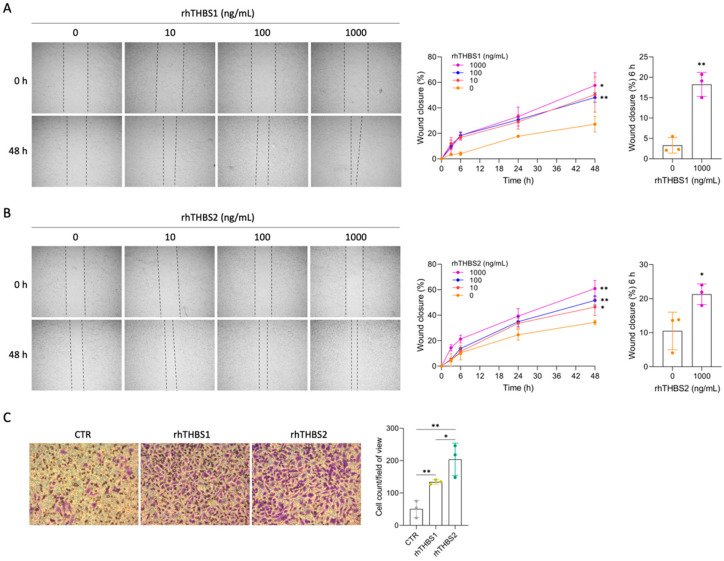
rhTHBS1 and rhTHBS2 increase CCLP1 cell migration and invasion. CCLP1 cells were subjected to a scratch assay and then treated with rhTHBS1 (**A**) and rhTHBS2 (**B**) at the indicated concentrations for 48 h; magnification 4×. The scratch assay wound closure rate was measured at the indicated times. (**C**) Trans-well invasion assay of CCLP1 cells in response to 1000 ng/mL of rhTHBS1 and rhTHBS2. Bar graph of trans-well invasion assay represents the mean value ± SD from independent experiments performed in triplicate, magnification 20×; * *p* < 0.05; ** *p* < 0.01 (*t*-test).

The effect of increasing concentrations of rhTHBS1 and rhTHBS2 on cell migration was also investigated. The wound-healing assay revealed that both rhTHBS1 and rhTHBS2 increased CCLP1 migration in a dose-dependent manner (Figure 3A,B).

Notably, for both the thrombospondins, the treatments at the 1000 ng/mL dose significantly increased CCLP1 cell migration after 6 h of incubation. Therefore, we used this concentration to assess the chemo-attractant effect of rhTHBS1 and rhTHBS2 on the invasiveness of the CCLP1 cells in trans-well invasion assays. The results showed that both the rhTHBS1 and rhTHBS2 increased the invasive abilities of CCLP1 cells (Figure 3C). Notably, the number of invasive cells after the rhTHBS2 treatment was significantly higher than that observed after the rhTHBS1 dose.

### 2.3. rhTHBS1 and rhTHBS2 Enhance the HuCCT-1 Malignant Phenotype

**Figure 4:**
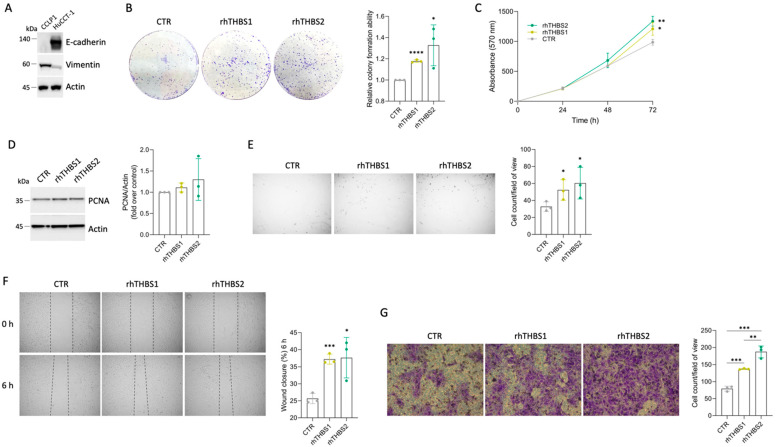
rhTHBS1 and rhTHBS2 promote growth, adhesion, migration, and invasion of HuCCT1 cells. (**A**) Representative Western blot analysis for E-cadherin and vimentin on whole-cell extracts of CCLP1 and HuCCT-1; actin was used as a loading control. (**B**) Clonogenic assays were performed with HuCCT-1 cells in response to 1000 ng/mL of rhTHBS1 and rhTHBS2. (**C**) Time course of HuCCT-1 cell growth assessed using the MTT cell viability assay at various time points in the presence of 1000 ng/mL of rhTHBS1 and rhTHBS2. (**D**) Western blot analysis for PCNA on whole-cell extracts of HuCCT-1 cells cultured in the presence of 1000 ng/mL of rhTHBS1 and rhTHBS2 for 48 h; actin was used as a loading control. Quantification from three independent immunoblots by densitometry analysis is shown. (**E**) Impact on cellular attachment in HuCCT-1 cells in response to 1000 ng/mL of rhTHBS1 and rhTHBS2; magnification 4×. (**F**) HuCCT-1 cells were subjected to a scratch assay and then treated with 1000 ng/mL of rhTHBS1 and rhTHBS2. The scratch assay wound closure rate was measured after 6 h of treatments; magnification 4×. (**G**) Trans-well invasion assay of HuCCT-1 cells in response to 1000 ng/mL of rhTHBS1 and rhTHBS2; magnification 20×. The bar graph of the trans-well invasion assay represents the mean value ± SD from independent experiments performed in triplicate; * *p* < 0.05; ** *p* < 0.01; *** *p* < 0.001; **** *p* < 0.0001 (*t*-test).

iCCA comprises two different subtypes, large- and small-duct iCCA, reflecting their anatomical location along the intrahepatic biliary tree [1,22]. Large-duct iCCA arises near the hepatic hilus proximal to right and left hepatic ducts, while small-duct iCCA shows a more peripheral location in the liver. In terms of cell composition, mucin-secreting cells are more common in large-duct iCCA compared to small-duct iCCA. HuCCT-1 is an established iCCA mucin-secreting cell line that expresses epithelial cell adhesion molecule (EpCAM), whereas the so-far-analyzed CCLP1 shows a mucin/EpCAM-negative phenotype [23]. By assessing the expression of E-cadherin and vimentin, we further demonstrated that HUCCT-1 and CCLP1 deeply differ in terms of epithelial–mesenchymal transitional features (Figure 4A).

**Figure 5:**
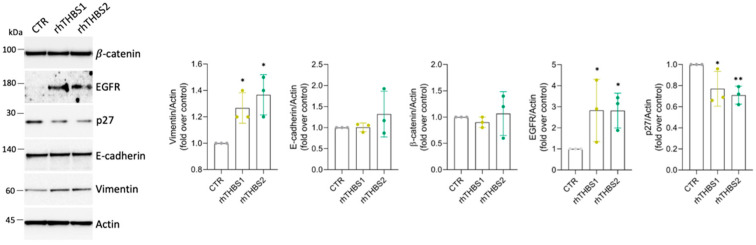
rhTHBS1 and rhTHBS2 commit HuCCT-1 cells to a mesenchymal phenotype. Western blot analysis for the indicated proteins on whole-cell extracts of HuCCT-1 cells cultured in the presence of 1000 ng/mL of rhTHBS1 and rhTHBS2 for 48 h; actin was used as the loading control. Quantification from three independent immunoblots by densitometry analysis is shown; * *p* < 0.05; ** *p* < 0.01 (*t*-test).

Thus, we wondered if the ability of the exogenous THBS1 and THBS2 to enhance the malignant phenotype of the iCCA cells is independent of the peculiar cell type. A slight increase in the ability to enhance colony formation was also observed in the HuCCT-1 cells (Figure 4B). Increased cell growth was further confirmed at 72 h by means of the MTT assay (Figure 4C), while no significant increases in PCNA expression were yet observed at 48 h (Figure 4D). rhTHBS1 and rhTHBS2 significantly increased cell adhesion, migration, and invasion in HuCCT-1 cells (Figure 4E–G). The effectiveness of rhTHBS2 in developing the HuCCT-1 malignant phenotype was higher than that observed with rhTHBS1. Finally, we tested the ability of the thrombospondins to commit HuCCT-1 cells to a mesenchymal phenotype. While we observed no changes in the expression of E-cadherin, the mesenchymal marker vimentin increased in cells treated with either rhTHBS1 or rhTHBS2 (Figure 5). EGFR and Wnt/β-catenin signaling are two of the pathways implicated in the oncogenesis and cancer progression induced by both THBS1 and THBS2, as well as in the activation of the EMT program [18,22]. Consequently, we investigated the expression levels of EGFR and β-catenin in HuCCT-1 cells following treatment with either rhTHBS1 or rhTHBS2. EGFR expression increased after treatment with both rhTHBS1 and rhTHBS2, while β-catenin expression remained unchanged (Figure 5). Moreover, the increased malignant phenotype suggested by the EGFR upregulation was further corroborated by the downregulation of the tumor suppressor p27. Overall, all these data indicate that THBS1 and THBS2 play a direct oncogenic role in both iCCA cell types.

## 3. Discussion

Understanding which paracrine mediators promote cancer cell proliferation and dissemination in the iCCA is crucial for identifying valuable therapeutic targets to counteract its progression and recurrence. We previously unveiled THBS1 and THBS2 functions in the angiogenesis/lymphangiogenesis switch [10]. They promote tumor-associated lymphangiogenesis, paving the way for the spreading of cancer cells into regional lymph nodes. Herein, we demonstrated that in iCCA, THBS1 and THBS2 have also a direct role in enhancing iCCA cells’ malignant phenotype by increasing cell proliferation, motility, invasion, and adhesion.

As secreted proteins, THBS1 and THBS2 abundance in the TME depends on both tumor and stromal cell expression. In the iCCA TME, we previously demonstrated that THBS1 and THBS2 are mainly expressed and released by CAFs and only to a lesser degree by tumor cells [10]. Although a role in developing a tumor cell phenotype by the autocrine positive feedback of thrombospondin expressed in the tumor cells may not be excluded, it is conceivable that these proteins exert their functions mostly by CAF-mediated paracrine action on tumor cells. Consequently, in this study, two different iCCA cell lines have been subjected to exogenously administered recombinant thrombospondins to assess the effects on cell behavior. Particularly, to evaluate the specific role of each single thrombospondin in iCCA pathobiology, we treated iCCA cells with either rhTHBS1 or rhTHBS2 individually. Results obtained after treatment with either rhTHBS1 or rhTHBS2 on CCLP1 and HuCCT-1 cells showed a significantly increased rate of colony formation ability and cell growth in both iCCA cell types, although with different intensities, with rhTHBS2 acting at a much lower concentration than rhTHBS1. This difference is reflected in human serum where THBS2 concentration is 100 ng/mL, whereas THBS1 is higher by an order of magnitude [10]. To date, the role of THBS1 and THBS2 in cancer cell growth has been only partially addressed with conflicting results. As an extracellular matrix (ECM) component, THBS1 has been reported to inhibit cell proliferation in small-cell lung carcinoma cells [24]. Similarly, in an in vivo model of lung adenoma, THBS1 expression decreased proliferation and promoted apoptosis [25]. On the contrary, THBS2 plays a role in promoting cell proliferation both in gastric cancer and uveal melanoma cells through the modulation of the PI3k-Akt signaling pathway [26,27]. In cancer, the divergent functions of thrombospondins in mediating protective or tumor-promoting effects depend on which thrombospondin receptor is expressed by cancer cells [14]. Both THBS1 and THBS2 share the TSR2 domain that interacts with the epidermal growth factor receptor (EGFR), which in turn is known to activate the PI3K/Akt/mTOR downstream signal pathway, known to be involved in cancer cell growth and survival [11,28]. Thus, since iCCA cells exhibit a sustained activation of EGFR and EGFR signaling plays a well-established role in the pathogenesis and progression of the disease [29]; in this respect, we found that both the thrombospondins were able to upregulate EGFR expression; it is conceivable that THBS1- and THBS2-induced iCCA cell growth may depend on TSR2s engaging EGFR.

In iCCA progression, the tendency to metastasize is a gradual and progressive event in which malignant cholangiocytes acquire a mesenchymal-like phenotype showing increased mobility and the ability to perform new adhesive interactions with the ECM and endothelial cells [30]. Here, we collected data showing the dose-dependent effects of both recombinant thrombospondins in increasing iCCA cell adhesion and motility. Despite THBS1 and THBS2 possibly having variable effects on tumor cell adhesion and migration, in the TME, they often behave as adhesive and chemotactic proteins by binding several members of the integrin family through the TSR1s, TSR12s, TSR3s, and the N-terminal domains [14]. In breast cancer, THBS1 promotes cancer cell adhesion and migration by engaging the α3β1 integrin [31]. Moreover, melanoma cell spreading and adhesion are promoted by the cooperation between αvβ3 integrin and the TSR1s domain of THBS1 [32]. In pancreatic ductal adenocarcinoma, the αvβ3 integrin and CD36 mediated cell growth and adhesion by binding THBS2 [33]. In the frame of iCCA, both α3β1 and αvβ3 integrins are highly expressed in tumor cells [34,35]. Most importantly, the inhibition of presentation on the cell surface of the integrin αvβ3 was associated with decreased cell adhesion and migration in iCCA [35]. All these considerations suggest a possible role of α3β1 and αvβ3 integrins in the THBS1- and THBS2-mediated enhancement of iCCA cell malignant behavior and open future scenarios for further investigation on this topic.

We also demonstrated that both rhTHBS1 and rhTHBS2 significantly increased both iCCA cells’ invasiveness. Interestingly, in both CCLP1 and HuCCT-1 cells, we observed that invasiveness induced by rhTHBS2 was higher than that measured after rhTHBS1 treatment. Similarly, we observed a greater capacity of rhTHBS2 to enhance the malignant behavior of the iCCA cells in committing the epithelial HuCCT-1 cell line to a mesenchymal-like phenotype. Since THBS1 and THBS2 showed different intensities in enhancing colony formation and cell adhesion, and even though they equally induced EGFR overexpression, it could be conceivable that they may also regulate iCCA cell invasiveness by activating alternative signaling pathways. In support of this hypothesis, in breast cancer, THBS2 was found to promote cell invasion by activating the PI3K/AKT signaling pathway [36], while THBS1-induced breast cancer cell invasion occurs by the YAP/FAK and transforming growth factor (TGF)-beta/Smad3 axes [37,38]. Further investigations are needed to gain insights into the signaling pathways driving THBS1- and THBS2-induced iCCA cell invasion.

THBS1 and THBS2 were found to be upregulated in the majority of tumoral iCCA samples, though not universally across all iCCA patients [10]. In a recent large-scale proteogenomic study, four iCCA subgroups (S1–S4) were identified, each exhibiting unique biological and clinical features and classified based on their malignant behavior [39]. THBS1 and THBS2 were enriched in the patients of the S2 subgroup, who were characterized by higher lymph node metastasis and lower overall survival. Furthermore, a single-cell transcriptomic analysis involving 14 pairs of iCCA tumors and non-tumor liver tissues classified iCCA into two subtypes according to the expression of S100P and SPP1 [40]. THBS2 expression was aberrant in the S100P+ SPP1− iCCA subgroup, associated with a worse overall survival score. These findings collectively suggest a possible prognostic value for THBS1 and, particularly, for THBS2, emphasizing the significance of our results regarding the direct role of these proteins on the iCCA cells. Moreover, decreased p27 expression is associated with aggressive tumor behavior and worse prognosis in iCCA patients. Thus, our data indicating p27 downregulation further confirm the pro-oncogenic role of both THBS1 and THBS2 in the context of iCCA [41,42].

In conclusion, we provided data showing that the role of THBS1 and THBS2 is not only to hinder the vascular network and promote tumor-associated lymphangiogenesis but also to exacerbate the malignant behavior of the two iCCA cell lines that matched the recent classification of iCCA in small (i.e., non-mucin-producing glands) and large (i.e., mucin-producing glands) subtypes [22]. The increase in the malignant phenotype of the iCCA cells by treatment with recombinant thrombospondins alone suggested that no additional factors are involved in its pathological progression. Many issues on the role of THBS1 and THBS2 in iCCA progression remain to be addressed: iCCA cell receptors engaged by THBS1 and THBS2; the signaling pathways underlying the role of both the thrombospondins in iCCA cell growth and spreading; and if additional roles unique to each thrombospondin are still missing. The data presented here strongly suggest the need to target THBS1 and THBS2 to counteract the iCCA progression and will support future in vitro and in vivo studies in addressing these issues.

## 4. Materials and Methods

### 4.1. Reagents

All reagents were from Sigma (St. Louis, MO, USA) unless stated otherwise.

### 4.2. Cell Culture

The CCLP-1 and HuCCT-1 human iCCA cell lines used in this study were kindly provided by Prof. D. Alvaro (Sapienza University of Rome) and F. Marra (University of Florence), respectively. Cells were maintained in RPMI 1640 medium containing 10% fetal bovine serum (FBS) and 1% penicillin–streptomycin–glutamine (Gibco/BRL; Life Technologies) at 37 °C in a humidified atmosphere of 5% CO_2_ in air. Human recombinant THBS1 and THBS2 (rhTHBS1 and rhTHBS2) were purchased from R&D Systems Europe Ltd. (Abingdon, Oxfordshire, UK) and cellular functional assays were performed at doses of 10 ng/mL, 100 ng/mL, and 1000 ng/mL versus control medium. 

### 4.3. Clonogenic Assay and Viable Cell Counting

A total of 10^3^ cells/well were plated in 6-well dishes, exposed to treatments, and incubated at 37 °C. One week later, cell colonies were fixed, stained with methanol 25% and crystal violet 0.1% (Santa Cruz Biotechnology, Dallas, TX, USA, sc-207460), and counted using the ZEN 2.0 software (Carl Zeiss, Oberkochen, Germany). Trypan blue stain and the hemocytometer were used to determine viable cell numbers. Cell viability was also measured using the colorimetric 3-(4,5)dimethylthiazol-2-yl)-2,5-diphenyltetrazolium bromide (MTT) assay kit (CyQUANT™ MTT Cell Viability Assay V13154, Invitrogen, Thermo Fisher Scientific, Waltham, MA, USA). Cells were grown in 96-well plates and incubated with 1000 ng/mL of rhTHBS1 and rhTHBS2 for 24 h, 48 h, and 72 h. Control cells were prepared in plates containing only medium. At the end of the incubation period, MTT was added to each well and incubation was carried out for 4 h at 37 °C. Living cells reduced MTT to formazan, which was quantified by measuring the absorbance at 570 nm (Multiskan™ FC Microplate Photometer, Thermo Fisher Scientific, Waltham, MA, USA).

### 4.4. In Vitro Adhesion Assay

Cells were seeded into Matrigel-coated (Corning^®^ Matrigel^®^ Growth Factor Reduced (GFR) Basement Membrane Matrix, Corning, NY, USA) wells (5 μg/well) in a 24-well plate at a seeding density of 5 × 10^4^ cells per well. Cells were exposed to treatments at 37 °C in a humidified atmosphere of 5% CO_2_ in air. Following 45 min of incubation, the plate was shaken at 500 rpm for 15 s and non-adherent cells were washed off with phosphate-buffered saline (PBS). The remaining cells were fixed and stained with crystal violet as previously described. The number of adherent cells was evaluated by using the ZEN 2.0 software. For field counting, at least four images were taken for each replicate in a light microscope with a 4× objective. For each image, the total number of cells was counted, and the average was calculated. The data are shown as the mean number of cells per visual field (magnification ×4) of at least 3 replicate wells.

### 4.5. In Vitro Wound Healing Assay

Cells were grown to confluence on 12-well plates in the complete medium as previously described. Once at confluence, cells were starved overnight in RPMI 1640 medium supplemented with 0.2% FBS. A linear scratch was made using a sterile 200 μL pipette tip and cells were always exposed to treatments in low-FBS medium. Images of the scratch area were captured immediately after the scratch and at 6, 24, and 48 h using the Zeiss AxioCam ERc 5s digital camera on a Zeiss Primovert light microscope (Carl Zeiss, Oberkochen, Germany). The remaining wound area was measured using ImageJ software 1.53k (National Institutes of Health, Bethesda, MD, USA) and normalized to time 0 wounds.

### 4.6. In Vitro Invasion Assay

Invasion assays were performed using a trans-well system (ThinCert™ 8-µm pore size, Greiner bio-one, Vilvoorde, Belgium). Briefly, each chamber (24-well insert) was pre-coated with 60 μL of 0.5 μg/μL Matrigel™ and 2 × 10^5^ cells were plated on the apical side of the chamber in low-FBS medium (RPMI 1640 medium supplemented with 0.2% FBS). RPMI supplemented with 10% FBS and the recombinant thrombospondins as chemoattractants were added to the basal compartment. The cells were allowed to migrate for 48 h, and then, the cells that remained on the apical side of the chamber were gently scraped off using wetted cotton swabs. The cells that had migrated to the basal side were fixed and stained with crystal violet and photographed. The data are shown as the mean of the cell counts of 6 visual fields (magnification ×20) of 3 replicate wells.

### 4.7. Immunoblotting

A total of 10 μg of protein was loaded and separated in NuPAGE™ 4–12% Bis-Tris Mini by SDS-PAGE with MOPS running buffer and electroblotted onto nitrocellulose membranes (Amersham Protan 0.45 µm cod. 10600041 GE, Healthcare, Little Chalfont, UK). The blots were incubated with primary and secondary antibodies. The antibodies were revealed using Luminata^TM^ Western HRP Chemiluminescence Substrates (Millipore Corporation, Billerica, MA, USA). To control for equal protein loading and transfer, the membranes were stained with Ponceau S solution. The following primary antibodies were used: anti-Actin (Santa Cruz Biotechnology, Dallas, TX, USA, sc-1615) at a dilution of 1:1000, anti-PCNA (Cell Signaling, Danvers, MA, USA, #13110) at a dilution of 1:1000, anti-PARP (Cell Signaling, Danvers, MA, USA, #9532) at a dilution of 1:1000, anti-Vimentin (Cell Signaling, Danvers, MA, USA, #5741) at a dilution of 1:1000, anti-E-cadherin (BD Biosciences, Franklin Lakes, NJ, USA, 610181) at a dilution of 1:200, anti-EGFR (Sigma-Aldrich, St. Louis, MO, USA, 05-104) at a dilution of 1:1000, anti-β-catenin (BD Biosciences, NJ, USA, 610153) at a dilution of 1:500, and anti-p27 (Santa Cruz Biotechnology, Dallas, TX, USA, sc-528) at a dilution of 1:200. The secondary peroxidase-conjugated antibodies were from Jackson ImmunoResearch (Cambridge House, Ely, UK) and used at a 1:5000 concentration. The chemiluminescent blots were imaged with the ChemiDoc^TM^ Touch Imaging System (Bio-Rad Laboratories, Hercules, CA, USA). Band densities were quantified by ImageLab software version 5.1.2 (Bio-Rad).

### 4.8. Statistical Analysis

GraphPad Prism version 8 software (GraphPad Software, Boston, MA, USA, www.graphpad.com, accessed on 30 January 2019) was used to undertake the statistical analysis. Data are shown as mean ± standard deviation (SD) and were collected from at least three separate studies. Student’s *t*-test analysis was used to examine two separate group comparisons. Statistical significance was set at *p* < 0.05.

## Data Availability

Data are contained within the article.
